# Exercise preference in stroke survivors: a concept analysis

**DOI:** 10.3389/fneur.2024.1326649

**Published:** 2024-02-13

**Authors:** Yuting Dai, Huiling Shi, Kangling Ji, Yuxin Han, Minerva De Ala, Qing Wang

**Affiliations:** ^1^Department of Neurology, Nanjing Drum Tower Hospital, Affiliated Hospital of Medical School, Nanjing University, Nanjing, China; ^2^School of Nursing, Nanjing University of Chinese Medicine, Nanjing, China; ^3^School of Nursing, Philippine Women’s University, Manila, Philippines; ^4^Department of Nursing, Nanjing Drum Tower Hospital, Affiliated Hospital of Medical School, Nanjing University, Nanjing, China

**Keywords:** stroke, exercise, rehabilitation, patient preference, concept analysis

## Abstract

**Background:**

Exercise preference in stroke survivors is related to their adherence to long-term rehabilitation regimen and functional recovery. Although explored recently, the term exercise preference still lacks a clear definition.

**Objective:**

The aim of this study is to conceptualize exercise preference in stroke survivors.

**Methods:**

The Walker and Avant method was applied as a framework for the conceptual analysis of exercise preference. Data from 34 publications were collected using seven databases (PubMed, Web of Science, Embase, CINAHL, CNKI, Wanfang Data, and CBM) and applied in the analysis. The search period was from the inception of the database to April 30, 2023.

**Results:**

Exercise preference in stroke survivors was defined according to four attributes: priority of choice, behavioral tendency, affective priming, and patience in adherence. The common antecedents of the concept of exercise preference in stroke survivors were classified into patient-related, therapy-related, and environmental-related categories and the consequences were classified into three categories: patient-related, rehabilitation provider–related, and rehabilitation service system–related.

**Conclusion:**

Exercise preference in stroke survivors refers to the patient’s choice, tendency, affective response, and attitude toward engagement in the recommended rehabilitation regimen. It is beneficial for understanding the essential attributes of exercise preference in stroke survivors by clarifying the concept. In addition, it will facilitate the development of instruments for assessing exercise preference in stroke survivors and the construction of theory-based intervention programs that can improve adherence to exercise rehabilitation.

## 1 Introduction

Stroke has become a major global public health problem, and loss of motor function is the main cause of patient disability. More than 80% of the stroke survivors have varying degrees of motor function loss, and approximately 50% of them experience motor dysfunction in 3 months after stroke onset, leading to dependence, limited mobility, and “hard return” (1, 2). The extreme challenge in motor recovery is to minimize motor impairment. Rehabilitation is key to promoting motor recovery after a stroke, and much of the rehabilitation experience—whether inpatient, outpatient, or at home—as it relates to motor recovery after acute care discharge revolves around physical activity and exercise (3). However, motor recovery involves actively acquiring knowledge and skills through external support to promote physical, psychological, and social function improvement. Rehabilitation is a complex behavior regulated by automatic process (habit) and goal-directed control process (intention) (4, 5), and patient preference plays a crucial role in motivation and control (6). Therefore, exercise preferences in the rehabilitation process of stroke survivors, which will be closely related to their adherence to long-term rehabilitation processes and functional recovery, need to be studied.

Evidence suggests stroke patients may benefit more from earlier, more intensive rehabilitation (7, 8). Exercise is an integral component of rehabilitation after stroke onset and plays a crucial role in promoting functional independence (9). Exercise rehabilitation is a long-term, dynamic, multifactorial, and complex process, and stroke survivors present different exercise preferences over time (10). Patients want themselves, rather than the techniques, to be kept as the center of attention in the rehabilitation process, such as the exercise mode they are interested in, accessible, positive, and meaningful as they perceived it, confidence, external supervision and support, and so on (11–13). Exercise regimes need to be designed that keeping in mind exercise preferences that promote the affective response of stroke survivors so that they become engaged and motivated to be physically active (14, 15).

The term preference has been used for a long time in the fields of psychology and economics, which refers to weighing the risk and benefit for the individual (16, 17). It has mainly been studied in personal financial investment decisions, also reflecting choice tendency or behavioral intention (17, 18). As the patient-centered care was proposed, patient preference has been valued and recognized as a crucial element of best nursing practice (19). Patient preference (PP) information is defined as qualitative or quantitative assessments of the relative desirability or acceptability of patients among specified alternatives or choices among outcomes or other attributes that differ among alternative health interventions (20). However, we cannot simply equate the concept of patient preference information with the concept of exercise preference in stroke survivors. The concept of exercise preference in stroke survivors is more focused on the personal preferences in the recommended rehabilitation regimen. Exercise preference in stroke survivors has been presented only as a fragmented information in several studies (13–15). But exercise preference of stroke survivors has not been extensively covered, including the heterogeneity of personal values and inclination for long-term rehabilitation (11, 13, 21).

Clarifying the concept of exercise preference in stroke survivors in the rehabilitation process can provide primary knowledge about preferences in rehabilitation in the selected population with similar traits and ensure consistency in its utilization and adoption. It can also enable scholars and researchers to develop assessment instruments for evaluating the exercise preference of stroke survivors and construct hypotheses that clearly mirror the relationships between and among the related concepts and factors. Furthermore, health care professionals designing the rehabilitation regimes for stroke survivors could adopt personalized strategies that can support adherence to exercise and improve self-management of the rehabilitation process. Hence, this study aims to provide a clear and evidence-based definition of exercise preference in patients with stroke and construct its antecedents, attributes, consequences, and empirical referents.

## 2 Materials and methods

### 2.1 The concept analysis approach

The Walker and Avant’s method of analysis was be adopted to evaluate the concept of exercise preference in stroke survivors, which contained the following eight steps (22): (1) select a concept (2), determine the aims or purposes of analysis, (3) identify all the uses of the concept that you can discover, (4) determine the defining attributes (5), identify a model case (6), identify borderline, related, contrary, invented, and illegitimate cases (7), identify antecedents and consequences, and (8) define empirical referents.

### 2.2 Data sources

A comprehensive and broad search for the term “exercise preference of stroke survivors” was performed in PubMed, Web of Science, Embase, CINAHL, China National Knowledge Infrastructure (CNKI), Wanfang Data, and China Biology Medicine disc (CBM) from their inception to April 30, 2023 to confirm the basic elements of the concept. The keywords “stroke,” “apoplexy,” “cerebrovascular accident,” “cerebrovascular apoplexy,” “CVA,” “rehabilitation,” “exercise rehabilitation,” “exercise,” “physical activity,” “preference,” “value,” “favor” were used as search terms individually or in combination with each other. The specific search strategy is shown in Supplementary material S1. In addition, references from the retrieved literature (especially review literature) were also reviewed to supplement and ensure a complete search. The inclusion criteria involved literature in English or Chinese, full-text publications, and academic journals that can confirm the concept of exercise preference using stroke survivors as a sample. Commentaries, editorials, and dissertations were excluded.

**Figure 1:**
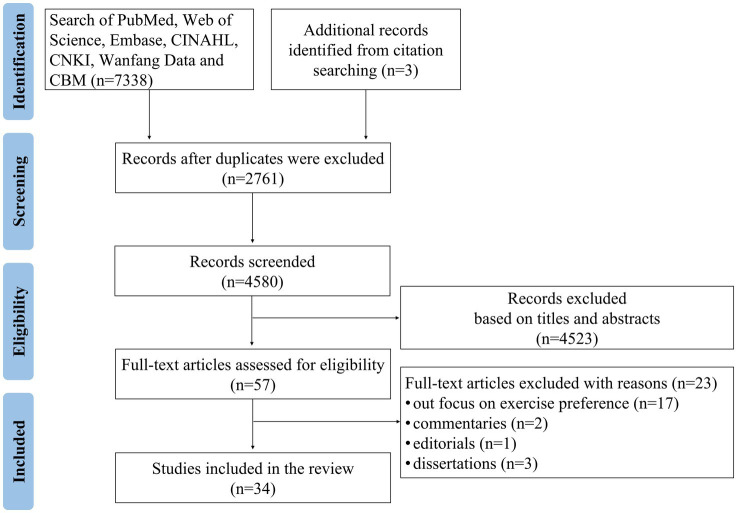
Flow diagram of the study selection.

A total of 34 studies with full-text publications were included in this study (Figure 1). The process of the search conforms to the Preferred Reporting Items for Systematic Reviews and Meta-Analyses (PRISMA) search strategy guidelines.

### 2.3 Data collection and management

Thirty-four studies were reviewed for systematic data collection and management, including study characteristics, as well as antecedents, attributes, and consequences of concepts. The information was extracted and tabulated (Table 1). Clear definitions of exercise preference in stroke survivors are relatively rare in the aforementioned studies. Thus, only some studies include all the antecedents, attributes, and consequences of exercise preference in stroke survivors.

**Table 1:** Antecedents, attributes and consequences of exercise preference in stroke survivors.

Author	Year	Study design	Sample size	Antecedents	Attributes	Consequences
Abrahamson and Wilson	2019	Multiple case study design	74	/	Preferring exercises in controlled environments	/
Banks et al.	2012	Cross-sectional study	64	/	The choice of exercise with others, degree of structure of exercise program, independence, exercise location, and intensity	/
Bastos et al.	2021	Cross-sectional study	24	Motivation	Preferring exercises in controlled environments (gyms/rehabilitation centers) and those offered in groups (with family/friends, other individuals of similar age and health condition)	Personalized regimens providing; promoted the impeccable stroke rehabilitation service system construction
Bernhardt et al.	2020	Systematic review	/	/	/	Promoted the impeccable stroke rehabilitation service system construction
Blennerhassett et al.	2022	Mixed methods research	42	Capacity of mobility; cognitive and emotional status; self-efficacy; personality traits; painful; fatigue; financial and social support; convenience and accessibility	The choice of exercise programs such as location	Enhanced the level of physical activity; personalized regimens providing; promoted the impeccable stroke rehabilitation service system construction; decreased in costs; improved rehabilitation services
Bonifacio et al.	2022	Systematic review	/	/	/	Promoted motor recovery; promoted quality of life of individuals with stroke
Bonner et al.	2016	Cross-sectional study	134	Capacity of mobility; cognitive and emotional status; self-efficacy; personality traits; painful; fatigue; financial and social support; convenience and accessibility	The choice of exercise programs such as location	Improved adherence to rehabilitation
Caetano et al.	2020	Cross-sectional study	93	Self-efficacy; outcome expectancy	The intention of individuals with stroke engaging in exercise	Improved adherence to rehabilitation
Chen et al.	2020	Qualitative research	13	Motivation; diversity and individualized of exercise program; convenience and accessibility	Pros and cons to weigh in this process	/
Forgea and Lorenz	2021	Systematic review	/	Capacity of mobility; cognitive and emotional status; motivation; financial and social support	/	/
Gard et al.	2019	Qualitative research	20	/	Choosing or refusing a certain mode of exercise depend on what they like or what they do not like when exercising	/
Geidl et al.	2018	Cross-sectional study	103	/	The choice of exercise programs such as social situation, location, type of exercise, intensity, frequency, and duration	/
Hunter et al.	2018	Cross-sectional study	176	Present bias	/	/
Jones et al.	2021	Mixed methods research	156	/	/	Improved the convenience and accessibility of rehabilitation delivery
Khoshbakht Pishkhani et al.	2019	Qualitative research	20	Capacity of mobility; beliefs in the benefits of exercise; perception of meaningful; financial and social support; family support; emotional stimulus	Choosing or refusing a certain mode of exercise depend on what they like or what they do not like when exercising; immediate gratification comes from the comfort of low intensity and lower-level exercise of physical inactivity or delayed gratification such as motor functional improvement by adherence to the recommended exercise	/
Ko et al.	2020	cohort study	168	/	/	Promoted motor recovery; promoted quality of life of individuals with stroke
Last et al.	2022	Qualitative research	11	Perception of meaningful; adaption; therapeutic relationships with therapists; diversity and individualized of exercise program; trust in rehabilitation providers; convenience and accessibility; behavior habit	A willingness to make effort on exercising to achieve a certain rehabilitation goal	Personalized regimens providing; job satisfaction; communication promoting in patients and rehabilitation team
Lin et al.	2022	Cross-sectional study	208	Capacity of mobility; education; previous experience and habit; financial and social support	/	/
Lin et al.	2021	Systematic review	/	/	/	Improved the convenience and accessibility of rehabilitation delivery
Luker et al.	2015	Systematic review	/	Preceding experience	Preferring the certain exercises as positive and meaningful to improve performance or linked to a better activity of daily life	/
Mahmood et al.	2022	RCT	52	Self-efficacy; education; painful	/	Improved adherence to rehabilitation; enhanced the level of physical activity; promoted motor recovery; promoted quality of life of individuals with stroke; personalized regimens providing; decreased in costs; improved rehabilitation services
Mameletzi et al.	2021	Systematic review	/	/	Choosing or refusing a certain mode of exercise depend on what they like or what they do not like when exercising	/
Matchar et al.	2022	RCT	266	Cognitive and emotional status; special incentives	/	/
Mohd Nordin et al.	2014	Qualitative research	23	/	Preferring exercises in controlled environments; as the stroke became chronic, motivation level declined	/
O’Dell et al.	2023	Systematic review	/	/	/	Promoted the impeccable stroke rehabilitation service system construction
Schuster-Amft et al.	2022	Single-arm clinical trial	14	Diversity and individualized of exercise program	/	/
Stark et al.	2019	Qualitative research	22	/	Preferring the certain exercises as positive and meaningful to improve performance or linked to a better activity of daily life	/
Temehy et al.	2022	Systematic review	/	/	/	Improved the convenience and accessibility of rehabilitation delivery
Timme et al.	2022	Cohort study	53	/	The affective response during exercise	/
Tyagi et al.	2018	Qualitative research	37	/	The intention of individuals with stroke engaging in exercise	/
Vadas et al.	2021	Systematic review	/	Self-efficacy; cognitive and emotional status; beliefs in the benefits of exercise; fatigue; adaption; therapeutic relationships with therapists; diversity and individualized of exercise program; trust in rehabilitation providers; financial and social support; family support; convenience and accessibility	Choosing or refusing a certain mode of exercise depend on what they like or what they do not like when exercising; preferring the certain exercises as positive and meaningful to improve performance or linked to a better activity of daily life; immediate gratification comes from the comfort of low intensity and lower-level exercise of physical inactivity or delayed gratification such as motor functional improvement by adherence to the recommended exercise	Improved adherence to Rehabilitation
Wijma et al.	2017	Systematic review	/	Therapeutic relationships with therapists; trust in rehabilitation providers	/	Professional identify; communication promoting in patients and rehabilitation team
Wu et al.	2001	Cross-sectional study	27	/	/	Improved adherence to rehabilitation
Yao et al.	2017	Cross-sectional study	98	/	Immediate gratification comes from the comfort of low intensity and lower-level exercise of physical inactivity or delayed gratification such as motor functional improvement by adherence to the recommended exercise	/

## 3 Results

### 3.1 Definitions and uses of concept

The common and universal understanding and usage of the term should be captured for a concept analysis. Based on the framework proposed by Walker and Avant (22) and synthesizing the 34 included studies, the concept of exercise preference in stroke survivors refers to the patient’s choice, tendency, affective response, and attitude toward engagement in the recommended rehabilitation regimen.

In 2001, Wu et al. (23) evaluated exercise preference for explaining the motor performance of the participants in performing a task and showed significant differences between the task preferences. In 2012, the Exercise Preference Questionnaire for stroke survivors was developed to capture exercise preferences and current exercise habits. The exercise preferences in stroke survivors contained five dimensions: (i) exercise with others, (ii) degree of structure of exercise program, (iii) independence, (iv) exercise location, and (v) intensity (24). In 2016, the Stroke Exercise Preference Inventory (SEPI) was constructed and consists of supervision support, confidence challenge, health and well-being, similar others, exercise context, home-alone, and music-TV (25). Furthermore, the concept of exercise preference has been developed both psychologically and theoretically. It addresses concerns that are relevant to people who require physical rehabilitation for conditions other than stroke. For medical professionals delivering exercise rehabilitation, especially in a home setting, the SEPI acts as a tool to promote specific discussion about choices and concerns and thus individualize the exercise regimes (14).

The studies mentioning concepts similar to exercise preference in stroke survivors have been found in the fields of nursing, medicine, psychology, physiology, and occupational therapy. Most of these are exploratory qualitative studies regarding experience (10), dilemma (26), expectation (27), facilitators, and barriers (11, 13, 26) in the rehabilitation process. Moreover, several quantitative studies have investigated the association between and among exercise preferences, potential barriers, and psychosocial factors such as self-efficacy and depression (14, 15, 21).

### 3.2 Attributes of exercise preference in stroke survivors

The exercise preference in stroke survivors were found to be the priority of choice, behavioral tendency, affective priming, and patience in adherence. Particulars of each attribute are described below.

#### 3.2.1 Priority of choice

Priority of choice is a crucial attribute of exercise preference in stroke survivors. This is mainly reflected in the choice of exercise programs such as social situation, location, type of exercise, intensity, frequency, and duration (28). Choosing or refusing a certain mode of exercise depends on what they like or what they do not like when exercising (13). Individuals with stroke are more interested in a certain exercise mode and more likely to make corresponding choices and exhibit better adherence (13, 29). Of course, there are also pros and cons to weigh in this process (30).

#### 3.2.2 Behavioral tendency

According to Caetano and colleagues (15), in the sphere of stroke rehabilitation, behavioral tendency reflects as the intention of individuals with stroke engaging in exercise. This often aligns with personal recovery goals and overall health goals. It usually presents as a willingness to make an effort to exercise to achieve a certain rehabilitation goal (26). As Bastos et al. (21) have observed, stroke survivors prefer exercises in controlled environments (gyms/rehabilitation centers) and those offered in groups (with family/friends, other individuals of similar age and health condition).

#### 3.2.3 Affective priming

An analysis of the preferences of the stroke survivors shows that when they perceived the certain exercises as positive and meaningful to improve performance or linked to a better activity of daily life, they will like it (10, 13). Affective priming was modulated by the valence of the preceding experience (10), behavior habit (26), emotional stimulus (29), and present bias (31), and exhibit the trait of dynamic, changeable, reversible, and personalized.

#### 3.2.4 Patience in adherence

It refers to the individual with stroke who is more concerned with immediate gratification that comes from the comfort of low-intensity and lower-level exercise of physical inactivity or prefers delayed gratification such as motor functional improvement by adherence to the recommended exercise (13, 29). As Bastos and colleagues have found, laziness leads to interrupting physical exercise (21). In other words, it means lack of patience in adherence to the exercise, and the stroke survivor is more likely to enjoy immediate gratification from “lying flat.”

### 3.3 Cases

#### 3.3.1 Model case

The model case is a real-life example involving all the defining attributes of the concept in a clinical context (22). A model case is the best example of concept application because it elaborates all of the defining attributes of the concept.

Mrs. Zhao, 62 years old, was suddenly affected by a stroke and was paralyzed on her left side. The rehabilitation regime was formulated before she was discharged as an acute inpatient. She engaged in the discussion actively and told the health professionals what exercises she prefers, such as walking, dancing, and swimming. She likes exercising in the park with other old friends, especially “square dancing.” She does not like high-intensity exercise. As proposed by the rehabilitation facility and specialty health institutions, she could not accept intense exercises because of previous bad experiences, distance, and financial reasons. Based on the physical functional recovery, she likes to design short-term goals. She is more interested in new rehabilitation approaches and techniques and willing to accept the challenge of higher goals. She thinks they are all beneficial to enhancing the performance. Regarding adherence to exercise, she expressed nothing could interrupt exercise and she will not be lazy. Then the professionals tailored an individualized rehabilitation regimen and supervision schedule for her. This personalized program included her exercise preferences and had a satisfactory efficacy of adherence and motor recovery.

The case of Mrs. Zhao contains all the previously discussed defining attributes of exercise preference in stroke survivors.

#### 3.3.2 Borderline case

Borderline cases are the scenarios that sit on the edge or boundary of a concept, making it difficult to definitively categorize. Those include most of the defining attributes, but one of them existing with a significant difference in time, intensity, or extent. Identifying borderline cases might reduce the ambiguity and inconsistencies between cases by clarifying attributes that are basic for the model (22).

Mr. Qian is 70 years old, has right hemiplegia and is living alone. Her right lower limb muscle strength was grade 2 at discharge. Walking or acquiring assistive devices to independently move were his main rehabilitation goals. After the acute care discharge, he was admitted to a rehabilitation facility. He has no particular preference for the exercise regimen. He faces various challenges in making decisions about his physical activity. He is open to trying various options but lacks strong motivation. He always wants to try the higher intensity exercises and repetitive exercise alone but falls into fatigue. He expressed the outcome expectation of motor recovery, but always felt lazy and unable to exercise. He lacks patience and prefers lying on the bed. Nursing staff paid more attention to him, including daily reminders, exercising in a direct supervision environment, and helping him visualize the restoration of motor function. After 2 weeks, he felt the guided exercises by professionals is effective to his motor recovery and perceived its meaningful outcome. Then he became active in exercising and his motor function gradually recovered, but the shoulder pain caused by high-intensity activities still affected him.

Mr. Qian’s case involves the attributes of uncertainty about the type of exercise he prefers. He recognizes the potential benefits of regular physical activity, and sometimes lacks the motivation to engage in structured exercise due to feelings of frustration and fatigue. He is willing to explore different exercise options, but lacks patience in adherence. There is a contradiction between behavioral tendency and patience in adherence. Therefore, nursing staff should adopt nudging strategies to help him achieve exercise goals.

#### 3.3.3 Related case

Related cases are instances that are in some way related to a concept but do not contain all the defining attributes. These help in understanding how the concept under study integrates into the network of concepts surrounding it (22).

Mrs. Sun is 52 years old and has right hemiplegia. Before stroke onset, she was a passionate “square dancing” lead dancer at their local community center square. After that, she was embarrassed by the disability caused by stroke and felt ashamed of meeting familiar people. In the initial phase, she refused to dance ever. She has been undergoing physiotherapy, and her therapist believes that dancing can be therapeutic if done with caution. Mrs. Sun modifies her dance mode to accommodate her post-stroke limitations. Instead of the fast-paced square dancing, she prefers slower dances like the ballroom dance, which requires less rapid movement and provides her with more stability. Her therapist introduced her to Tai Chi, emphasizing its benefits for balance and coordination, and she finds it meditative and beneficial, noticing that some of the movements remind her of dance.

The case of Mrs. Sun is related because it revolves around the attributes of priority of choice and behavioral tendency of a stroke survivor. Nevertheless, unlike the previous case where the individual was ambivalent about returning to a previous activity, Mrs. Sun actively seeks ways to adapt her zeal for dance to her current physical capabilities. The introduction of the exploration of Tai Chi as a complementary exercise adds layers to the concept of exercise preference in the population with stroke, emphasizing the affective priming and the potential for discovering new, beneficial activities post-stroke.

#### 3.3.4 Contrary case

The contrary case, as opposed to the model case, does not contain any of the main attributes of the concept (22). The clear example of not reflecting the concept is described below.

Mr. Li is 72 years old and is affected by left hemiplegia. She lives in the rural area alone. He has a son who is working in a neighboring city. He feels like an burden for his son and has no sense about the motor functional recovery. After acute care discharge, he was transferred to home. He refused to engage in any form of exercise despite being aware of its benefits for him. He has a strong aversion to physical activity and prefers a sedentary lifestyle, sometimes reading books and watching television. Nurses and therapists emphasized the importance of exercise for his recovery and overall health. However, despite having the physical capability to engage in light exercises like walking or stretching, he shows no interest. He often states that he never exercised before the stroke and he does not see why he should start now. The idea of exercising, even mildly, brings up fears of another stroke or injuring himself. He is more comfortable staying in his familiar, sedentary routine.

Unlike the primary concept where stroke survivors have some form of exercise preference (whether clear, ambivalent, or adapted), the case of Mr. Li displays a complete lack of interest in any physical activity as he harbors deep-seated fears or beliefs against exercise post-stroke. No attributes of exercise preference were included in this case. In the daily life activity of Mr. Li’s rehabilitation process, there is no choice, no effort to functional recovery, only the attitude of “1 day passes, 1 day counts.”

### 3.4 Antecedents and consequences

#### 3.4.1 Antecedents

Antecedents are incidents or events that must exist or occur prior to the concept’s occurrence (22). Concept occurrence is always preceded by antecedents. Those events that occur before the concept’s occurrence include patient-related antecedents, therapy-related antecedents, and environment-related antecedents. Patients’ related antecedent includes individuals’ capacity of mobility (11, 12, 14, 29), self-efficacy (9, 13–15), outcome expectancy (15), cognitive and emotional status (11, 13, 32), education (9, 12), beliefs in the benefits of exercise (13, 29), motivation (11, 21, 30), perception of meaningfulness (26, 29), pain (9), fatigue (13), personality traits (14), adaption (13, 26), and previous experience and habit (12). Therapy-related antecedents include therapeutic relationships with therapists (13, 26, 27), diversity and individualized exercise program (13, 26, 30, 33), and trust in rehabilitation providers (13, 26, 27). Environmental-related antecedents include financial and social support (11–13, 29), family support (13, 29), special incentives (32), and convenience and accessibility (13, 14, 26, 30).

#### 3.4.2 Consequences

Consequences are those events that occur as an outcome of concept’s occurrence (22). The consequences of exercise preference in stroke survivors are classified into the three categories: (i) patient-related, (ii) rehabilitation provider–related, and (iii) rehabilitation service system–related (3, 34, 35). Patient-related consequences include improved adherence to rehabilitation (9, 13, 15), enhanced the level of physical activity (9, 14), promoted motor recovery and quality of life of individuals with stroke (9, 36, 37). Rehabilitation provider–related consequences include providing a personalized regimen (9, 14, 21, 26), job satisfaction (26), professional identify (27), and communication promoting in patients and rehabilitation team (26, 27). Rehabilitation service system–related consequences include promoting the impeccable stroke rehabilitation service system construction (3, 14, 21), improved convenience and accessibility of rehabilitation delivery (34, 38, 39), and decreased in costs and improvement of rehabilitation services (9, 14).

**Figure 2:**
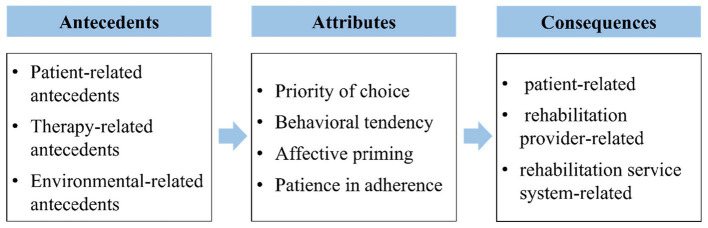
Conceptual structure of the exercise preference in stroke survivors.

The conceptual structure of the exercise preference in stroke survivors, including the relationships between antecedents, attributes, and consequences, is shown in Figure 2.

### 3.5 Empirical referents

The final step in concept analysis is defining empirical referents for the main attributes of the concept (22). Empirical referents are the categories of real phenomena that prove the occurrence of the concept itself. A problem must be proposed: How can I measure this concept or verify its existence in the real world? Empirical referents are the way in which defining attributes should be determined or assessed. They are not instruments for evaluating concepts (22). The purpose of the definitions of empirical referents is to accelerate the measurement of the concept, discern the concept, and to help the development of research instruments.

Exercise preference reflects value orientation and choice in the process of rehabilitation. Very few studies have been conducted on exercise preference in patients with stroke. Some of the studies only showed exercise preference as part of facilitators and barriers to engaging in exercise or rehabilitation (11, 13, 26), or has been proposed in formulating the rehabilitation regimes (9).

The attributes of exercise preference in stroke survivors are measured using the tools for evaluating post-stroke conditions. Exercise Preference Questionnaire (*stroke*) (24) consists of 33 questions that are divided into three sections designed to capture exercise preferences and current exercise habits. There are 22 questions regarding different exercise preferences, which contain the domains of exercise with others, degree of structure of exercise program, independence, exercise location, and exertion. The Stroke Exercise Preference Inventory (SEPI) (25) consists of 13 preference items and an optional 9-item module on barriers to exercise participation after stroke. There are 7 exercise preference factors including supervision support, confidence challenge, health and well-being, similar others, exercise context, Home-alone, and music-TV. SEPI is the first stroke-specific tool for evaluating exercise preference in rehabilitation process and can be used in rehabilitation research and practice. Such large individual differences in exercise preference have been found in Bonner’s investigation using SEPI, which may explain the poor uptake and adherence of one-size-fits-all exercise programs.

Based on literature review, the tools for measuring exercise preference in stroke survivors were more concerned with choice and behavior, focusing less on affective priming and patience in adherence. Exercise preference in stroke survivors is associated with not only their attitude and beliefs of patients but also emotional and psychological aspects. Therefore, attention to precise evaluation of this concept and construction of appropriate tools for stroke survivors become essential.

## 4 Discussion

There are four attributes as a result of the concept analysis derived from the critical literature review: (i) priority of choice, (ii) behavioral tendency, (iii) affective priming, and (iv) patience in adherence. We discuss the meaning and features of each attribute and analyze the antecedents and consequences in depth.

For individuals, engaging in exercise after a stroke is a long-term process that continues beyond hospital to discharge, highlighting the need to learn skills by stroke survivors and adhere to the prescribed rehabilitation regimen. The personalized exercise strategies have been effective in rehabilitation adherence and motor recovery (9, 29, 40). This means a specific exercise choice made by themselves based on what they like or more interested in (classified as “priority of choice” in this analysis). They are provided personalized education and supervision. Vadas et al. (13) showed that clinicians are advised to spend time learning about each individual’s life circumstances, so they can tailor proposed exercise programs to patients’ personal situations, preferences, and needs to have a positive effect on adherence. In an interpretive description qualitative study by Last et al. (26), the interviewed participants stated the following: “I enjoyed the therapy because I could have fun in there, and if the music was on I could dance … it was a happy place for me…,” supporting this attribute. Participants want to be involved in planning and setting goals, which integrate their choice in it (41), so they enjoy and are motivated to engage in physical exercises. People who engaged in an exercise rehabilitation that they did not like show poor adherence and fall into bad mood. “They wanted me to work with the silly putty stuff there … I found that it hurt me more to use it…” (26) Priority of choice is a critical point in the development of stroke rehabilitation regimen and can have a significant impact on adherence.

The second attribute, “behavioral tendency,” may emerge based on the “priority of choice” attribute. Task-specific exercise and repetition or mass practice is the critical principle of an effective exercise program for stroke survivors. How to design the task and exercise schedule to help them willing to be engaged and exercising? Individuals after suffering a stroke prefer exercising in controlled environments (21). In interviews described in the study by Mohd Nordin et al. (42) and Chen et al. (26), the participants gave the following statements: “I wanted to get moving because the physio was so good in hospital… but then when you come home there’s nothing…” “I enjoy giving clarification on how to do the exercises. …she would watch, give some little corrections…,” supporting this attribute. On the contrary, sometimes participants tend to refuse or lack motivation for a specific task of exercise. The interviews described in the study by Last et al. (26) and Tyagi et al. (43) show this: “… they got me to make a sandwich, use the toaster, make coffee—little things like that-they were not challenging at all for me.” “A lot of it is setting up the equipment that we do not want to do. I hate doing it …” Behavioral tendency plays an important role in long-term rehabilitation process, which determines individuals’ behavior intention in a certain exercise mode, including environments, supervision, and support. It also determines whom they want to exercise with, as well as if they want to engage in the exercise or adhere to the rehabilitation regimen (14, 15).

The affective response during exercise is an important factor for long-term exercise adherence (44). If the stroke survivors perceive the exercise or activity as positive and meaningful, they are more willing to perform that exercise or activity. This appeared as the third attribute, “affective priming.” Repeated experiences of core affective reactions (i.e., the degree to which exercise or rehabilitation feels useful or useless) during exercise in stroke survivors and automatic affective valuations would be generated, which influence their decision to engage in exercise behavior (13, 15). In an interview described in the study by Stark et al. (45): “The first time … I managed (it) once, toward the end of the study I managed (it) 15 times in 30 s. It was the highlight of the study.” “We had our fun and we were happy when things got better.” Patients were motivated when they subjectively experienced progress or their caregivers observed the progress. Nevertheless, as the automatic affective valuations are negative, they tend to drop out, as a participant mentioned in an interview by Last, et al.: “No, because … it’s not gonna do nothing for me. I’m not gonna get nothing out of it … I do not think it’s helping me” (26).

The last attribute of exercise preference in stroke survivors was “patience in adherence.” In the rehabilitation process, stroke survivors made a choice of exercise regimen that they like and accept. They engage in a series of activities with a sense of emotional involvement or commitment with a deliberate application of effort (11). As the stroke became chronic, their motivation level declined. Some of them act lazy, as described in a study by Mohd Nordin et al. (42) in focus group discussions: “Initially, I was motivated. After several months, I do not feel that excited anymore.” “I feeling lazy at home…” Bastos et al. (21) pointed out that laziness is the crucial factor that leads to interruption in physical exercise. Some of the patients with more severe disability caused by stroke also weighed their current efforts against future benefits, and thus choose to continue exercising or give up (46). More present-oriented individuals prefer an immediate, smaller reward, as observed by Forgea et al. (11) who reported that short-term conservative goals with an orientation toward physical functioning are preferred by stroke survivors. However, patients more concerned with their future were more likely to exhibit behaviors associated with positive recovery, as described by Chen et al. (30) in a qualitative study: “We wished to view their data in the long run.”

## 5 Limitations

Exercise rehabilitation after stroke is a long-term, dynamic, and complex process involving many factors. Exercise preference varies between stroke survivors and changes over time (10). Therefore, future research could focus on the different stages of stroke rehabilitation to better understand the exercise preference in stroke survivors and thus provide a more in-depth exploration of exercise rehabilitation after stroke.

## 6 Conclusion

This study presented an evidence-based and operational definition of exercise preference in stroke survivors and its antecedents, attributes, consequences, and empirical referents. It helped clarify the ambiguous concepts that are used in the context of exercise instead of preference. The operational definition of exercise preference in stroke survivors reflects the patient’s choice, tendency, affective response, and attitude toward engagement in the recommended rehabilitation regimen. Research regarding concept derivation of exercise preference in stroke survivors can help nurses and rehabilitation service providers to understand this concept better and pay more attention to it in the process of rehabilitation to promote individualized exercise regimen and enhance adherence to rehabilitation. In addition, this may facilitate the development of instruments for assessing exercise preference in stroke survivors and the construction of theory-based intervention programs that can improve adherence to exercise rehabilitation.

## Author contributions

YD: Conceptualization, Data curation, Formal analysis, Writing – original draft. HS: Formal analysis, Writing – review & editing. KJ: Formal analysis, Writing – review & editing. YH: Formal analysis, Writing – review & editing. MA: Writing – review & editing. QW: Conceptualization, Funding acquisition, Methodology, Writing – original draft, Writing – review & editing.
