# Supplementary material for: Exercise preference in stroke survivors: a concept analysis
Source: Front Neurol. 2024 Feb 13;15:1326649. doi: 10.3389/fneur.2024.1326649 (PMC10896848; doi:10.3389/fneur.2024.1326649)
Supplement: Supplementary file 1 [file Data_Sheet_1.docx]

**1.The search strategy for Pubmed**

(((stroke[MeSH Terms]) OR ("apoplexy"[Title/Abstract] OR "cerebral stroke"[Title/Abstract] OR "cerebrovascular stroke"[Title/Abstract] OR "cerebrovascular accident"[Title/Abstract] OR "cva"[Title/Abstract] OR "cerebrovascular apoplexy"[Title/Abstract] OR "brain vascular accident"[Title/Abstract] OR "cerebral infarction"[Title/Abstract] OR "cerebral ischemia"[Title/Abstract] OR "cerebral hemorrhage"[Title/Abstract] OR "cerebrovascular disease"[Title/Abstract] OR "cerebral embolism"[Title/Abstract])) AND ((rehabilitation[MeSH Terms]) OR (((("rehabilitation exercise" [Title/Abstract]) OR (mobilization[Title/Abstract])) OR (exercise[Title/Abstract])) OR ("physical activity" [Title/Abstract])))) AND (((preference[Title/Abstract]) OR (value[Title/Abstract])) OR (favor[Title/Abstract]))

**Filters**: **English, Chinese**

**Period: inception - 2022/10/31**

**2.The search strategy for Web of Science**

**Query #1** (TI=(stroke OR "apoplexy" OR "cerebral stroke" OR "cerebrovascular stroke" OR "cerebrovascular accident" OR "cva" OR "cerebrovascular apoplexy" OR "brain vascular accident" OR "cerebral infarction" OR "cerebral ischemia" OR "cerebral hemorrhage" OR "cerebrovascular disease" OR "cerebral embolism")) OR AB=(stroke OR "apoplexy" OR "cerebral stroke" OR "cerebrovascular stroke" OR "cerebrovascular accident" OR "cva" OR "cerebrovascular apoplexy" OR "brain vascular accident" OR "cerebral infarction" OR "cerebral ischemia" OR "cerebral hemorrhage" OR "cerebrovascular disease" OR "cerebral embolism")

**Query #2** (TI=(rehabilitation OR "rehabilitation exercise" OR mobilization OR exercise OR "physical activity")) OR AB=(rehabilitation OR "rehabilitation exercise" OR mobilization OR exercise OR "physical activity")

**Query #3** (TI=(preference OR value OR favor)) OR AB=(preference OR value OR favor)

**Query #4** #3 AND #2 AND #1

**Filters: English, Chinese**

**Period: inception -2022/10/31**

**3.The search strategy for Embase**

('cerebrovascular accident'/exp OR 'stroke':ab,ti OR 'apoplexy':ab,ti OR 'cerebral stroke':ab,ti OR 'cerebrovascular stroke':ab,ti OR 'cva':ab,ti OR 'cerebrovascular apoplexy':ab,ti OR 'brain vascular accident':ab,ti OR 'cerebral infarction' OR 'cerebral ischemia' OR 'cerebral hemorrhage':ab,ti OR 'cerebrovascular disease':ab,ti OR 'cerebral embolism':ab,ti) AND ('rehabilitation'/exp OR 'rehabilitation exercise':ab,ti OR 'mobilization':ab,ti OR 'exercise':ab,ti OR 'physical activity':ab,ti) AND ('preference':ab,ti OR 'value':ab,ti OR 'favor':ab,ti)

**Filters: English, Chinese**

**Period: inception -2022/10/31**

**4.The search strategy for CINAHL**

**S1** TI ( stroke OR "apoplexy" OR "cerebral stroke" OR "cerebrovascular stroke" OR "cerebrovascular accident" OR "cva" OR "cerebrovascular apoplexy" OR "brain vascular accident" OR "cerebral infarction" OR "cerebral ischemia" OR "cerebral hemorrhage" OR "cerebrovascular disease" OR "cerebral embolism" ) OR AB ( stroke OR "apoplexy" OR "cerebral stroke" OR "cerebrovascular stroke" OR "cerebrovascular accident" OR "cva" OR "cerebrovascular apoplexy" OR "brain vascular accident" OR "cerebral infarction" OR "cerebral ischemia" OR "cerebral hemorrhage" OR "cerebrovascular disease" OR "cerebral embolism" )

**S2** TI ( rehabilitation OR "rehabilitation exercise" OR mobilization OR exercise OR "physical activity" ) OR AB ( rehabilitation OR "rehabilitation exercise" OR mobilization OR exercise OR "physical activity" )

**S3** TI ( preference OR value OR favor ) OR AB ( preference OR value OR favor )

**S4** S1 AND S2 AND S3

**Filters: English, Chinese**

**Period: inception -2022/10/31**

**5.知网（CNKI）**

TKA =（'中风'+'卒中'+'脑血管意外'+'脑梗死'+'脑梗塞'+'脑栓塞'+'脑缺血'+'脑出血'+'脑血管疾病'）

and TKA =（'康复'+'康复运动'+'运动'+'锻炼'+'身体活动'+'体力活动'）and TKA =（'偏好'+'价值观'+'偏爱'）

发表时间：不限-2022/10/31

**6.万方（Wanfang Data）**

题名或关键词：(中风 or 卒中 or 脑血管意外 or 脑梗死 or 脑梗塞 or 脑栓塞 or 脑缺血 or 脑出血 or 脑血管疾病) AND 题名或关键词：(康复 or 康复运动 or 运动 or 锻炼 or 身体活动 or 体力活动) AND 题名或关键词：(偏好 or 价值观 or 偏爱)

发表时间：不限-2022/10/31

**7.中国生物医学文献数据库（CBM）**

(( "偏好"[标题] OR "价值观"[标题] OR "偏爱"[标题]) OR( "偏好"[摘要] OR "价值观"[摘要] OR "偏爱"[摘要])) AND (( "康复"[标题] OR "康复运动"[标题] OR "运动"[标题] OR "锻炼"[标题] OR "身体活动"[标题] OR "体力活动"[标题]) OR( "康复"[摘要] OR "康复运动"[摘要] OR "运动"[摘要] OR "锻炼"[摘要] OR "身体活动"[摘要] OR "体力活动"[摘要])) AND (( "中风"[标题] OR "卒中"[标题] OR "脑血管意外"[标题] OR "脑梗死"[标题] OR "脑梗塞"[标题] OR "脑栓塞"[标题] OR "脑缺血"[标题] OR "脑出血"[标题] OR "脑血管疾病"[标题]) OR( "中风"[摘要] OR "卒中"[摘要] OR "脑血管意外"[摘要] OR "脑梗死"[摘要] OR "脑梗塞"[摘要] OR "脑栓塞"[摘要] OR "脑缺血"[摘要] OR "脑出血"[摘要] OR "脑血管疾病"[摘要]))

发表时间：不限-2022/10/31
